# Epibiont hydroids on beachcast *Sargassum* in the Mexican Caribbean

**DOI:** 10.7717/peerj.9795

**Published:** 2020-08-24

**Authors:** María A. Mendoza-Becerril, Elisa Serviere-Zaragoza, Alejandra Mazariegos-Villarreal, Crisalejandra Rivera-Perez, Dale R. Calder, Erika F. Vázquez-Delfín, Yolanda Freile-Pelegrín, José Agüero, Daniel Robledo

**Affiliations:** 1 CONACyT, Centro de Investigaciones Biológicas del Noroeste, La Paz, Baja California Sur, Mexico; 2 Centro de Investigaciones Biológicas del Noroeste, La Paz, Baja California Sur, Mexico; 3 Department of Natural History, Royal Ontario Museum, Toronto, ON, Canada; 4 Royal British Columbia Museum, Victoria, BC, Canada; 5 Applied Phycology Laboratory, Cinvestav-Unidad Mérida, Mérida, Yucatán, Mexico; 6 Medusozoa México, La Paz, Baja California Sur, Mexico

**Keywords:** Caribbean Sea, Epibionts, Hydrozoa, Macroalgae, Medusozoa

## Abstract

Massive accumulations of pelagic species of *Sargassum* have generated recent social, economic and ecological problems along Caribbean shores. In the Mexican Caribbean, these events have prompted the study of diverse biological and ecological aspects of these macroalgae. However, studies on their associated biota, including Hydrozoa, remain scarce. This research provides important species observations in an area where data is lacking. The occurrence and percent cover of hydroids on *Sargassum* thalli collected on the beach at Puerto Morelos, Quintana Roo, Mexico from April 2018 to March 2019 was studied. Three pelagic species and morphotypes of *Sargassum* from this area were analyzed: *Sargassum fluitans* III, *S. natans* I and *S. natans* VIII, as well as a benthic species, *S. polyceratium* var. *ovatum*. A total of 14 taxa of hydroids, belonging to the superorders “Anthoathecata” and Leptothecata, were identified. In our study, more hydroid taxa were observed on axes of the different species of *Sargassum* than on leaves or aerocysts. In general, the greatest species richness of hydroids was observed from February to April. Results show that live hydrozoans attached to pelagic *Sargassum* are transported into the area. This should be considered in future management measures that address the recurring coastal abundance of *Sargassum* and its associated biota in the Caribbean region.

## Introduction

Hydroids are frequent epibionts on thalli of red, brown and green marine macroalgae (Oliveira & Marques, 2011). This is known to impact algal growth rates by reducing both their photosynthetic rates and the flexibility of their thalli, resulting in possible fragmentation (Niermann, 1986; Coelho-Souza et al., 2013). Macroalgal morphology, in addition to size and abundance, is considered an essential structural component in their habitat complexity, directly related to the species richness, abundance and diversity of associated invertebrate species assemblages (Hansen et al., 2011; Cunha, Maruyama & Jacobucci, 2018; Teagle et al., 2017; Pinna et al., 2020). Moreover, these attributes are also expressed in the distribution patterns of several epibiont species found in marine habitats (Heck & Wetstone, 1977; Tano et al., 2016) and their potential dispersion routes (Abé et al., 2013).

Of prime importance amongst invertebrate epibionts on species of the phaeophycean genus *Sargassum* are hydrozoans and bryozoans (Niermann, 1986; Morris & Mogelberg, 1973; Smith, Burns & Carpenter, 1973; Cunha & Jacobucci, 2010; Oliveira, Marques & Migotto, 2006; Oliveira & Marques, 2011). To date, the number of hydroid species (~50) occurring on benthic species of *Sargassum* (Nishihira, 1965; Shepherd & Watson, 1970; Morri & Bianchi, 1999; Haddad & Chiaverini, 2000; Cunha & Jacobucci, 2010) is higher than hydroid species (~18) growing on pelagic species of *Sargassum* (Parr, 1939; Ryland, 1974; Calder, 1995; Mendoza-Becerril, Simões & Genzano, 2018; Govindarajan et al., 2019). On thalli of pelagic *Sargassum*, 14 species of hydroids have been reported in the Sargasso Sea, offshore in the southern Gulf of Mexico, and in the tropical (excluding the Mexican Caribbean) and subtropical North Atlantic Ocean (Calder, 1995; Mendoza-Becerril, Simões & Genzano, 2018; Govindarajan et al., 2019), with five species being most frequent. However, in the Mexican Caribbean, no such studies have yet been undertaken, either for native or non-native species of *Sargassum*.

Since 2011, unusual biomass influxes of pelagic *Sargassum* have stranded on coastlines of the eastern Caribbean Sea and Gulf of Mexico (Gower, Young & King, 2013; Van Tussenbroek et al., 2017; Wang et al., 2019). This biomass is composed mainly of two pelagic species, *S. fluitans* (Bøergesen) Bøergesen and *S. natans* (Linnaeus) Gaillon, both having a broad geographic range (Parr, 1939; Butler et al., 1983). The two pelagic *Sargassum* species have distinct accepted morphotypes: *S. fluitans* III, X and *S. natans* I, II, VIII, IX (Parr, 1939). These accumulations have generated various social, economic and ecological problems in the Caribbean Sea, particularly for the tourism, healthcare, fishing, and wildlife sectors (Gower, Young & King, 2013; Maurer, De Neef & Stapleton, 2015; Hinds et al., 2016; Van Tussenbroek et al., 2017; Putman et al., 2018; Resiere et al., 2019; Rodríguez-Martínez et al., 2019, 2020). In 2015, large masses of these algae were reported on the coasts of Quintana Roo and Puerto Morelos, Mexico (Rodríguez-Martínez, Van Tussenbroek & Jordán-Dahlgren, 2016; SEMA, 2018). Such masses then decreased during 2016 and 2017 (Rodríguez-Martínez, Van Tussenbroek & Jordán-Dahlgren, 2016; Rodríguez-Martínez et al., 2019).

In 2018, massive strandings continued, however, reaching to Puerto Morelos (SEMA, 2018; Rodríguez-Martínez et al., 2019). Biological, ecological and chemical studies on these pelagic macroalgae have been carried out to understand and manage them (Milledge & Harvey, 2016; Cabanillas-Terán et al., 2019; Rosado-Espinosa et al., 2020). However, studies of their associated epibiota, including hydrozoans, remain scarce in the region.

Hydrozoans, with their complex life cycles (Russell, 1953; Gili & Hughes, 1995), versatility as substrate generalists (Calder, 1991a; Gravier-Bonnet & Bourmaud, 2006), and capacity for long-range transport, have been described as capable invaders that can create problems for the fishing and aquaculture industry (e.g., lesions on fish gills and skin, obstructions in the pipes of aquaculture installations, biofouling on aquaculture nets) (Wintzer et al., 2011; Baxter et al., 2012; González-Duarte et al., 2016). Some of these species have been recorded as epibiont hydroids of *Sargassum* (c.f. Calder, 1995; Oliveira, Marques & Migotto, 2006; Oliveira & Marques, 2011). Other negative impacts include their potential threat to public health and, by association, to local tourism. The toxins contained in the nematocysts of hydrozoans may produce stings and dermal lesions (Rifkin, Fenner & Williamson, 1993; Marques, Haddad & Migotto, 2002; Santhanam, 2020), including the nematocysts of hydroids on *Sargassum* (c.f. Oliveira, Marques & Migotto, 2006; Oliveira & Marques, 2011).

In this study, species of pelagic *Sargassum* stranding in Puerto Morelos (Mexican Caribbean) were analyzed to determine: (1) the species richness of epibiont hydroids; (2) the monthly/seasonal occurrence and percent coverage of hydroids on algae; (3) any differences in the presence of hydroids among axes, leaves and aerocysts of the algal thalli; (4) differences in composition of hydroids between *Sargassum* species and their morphotypes.

## Materials and Methods

**Figure 1:**
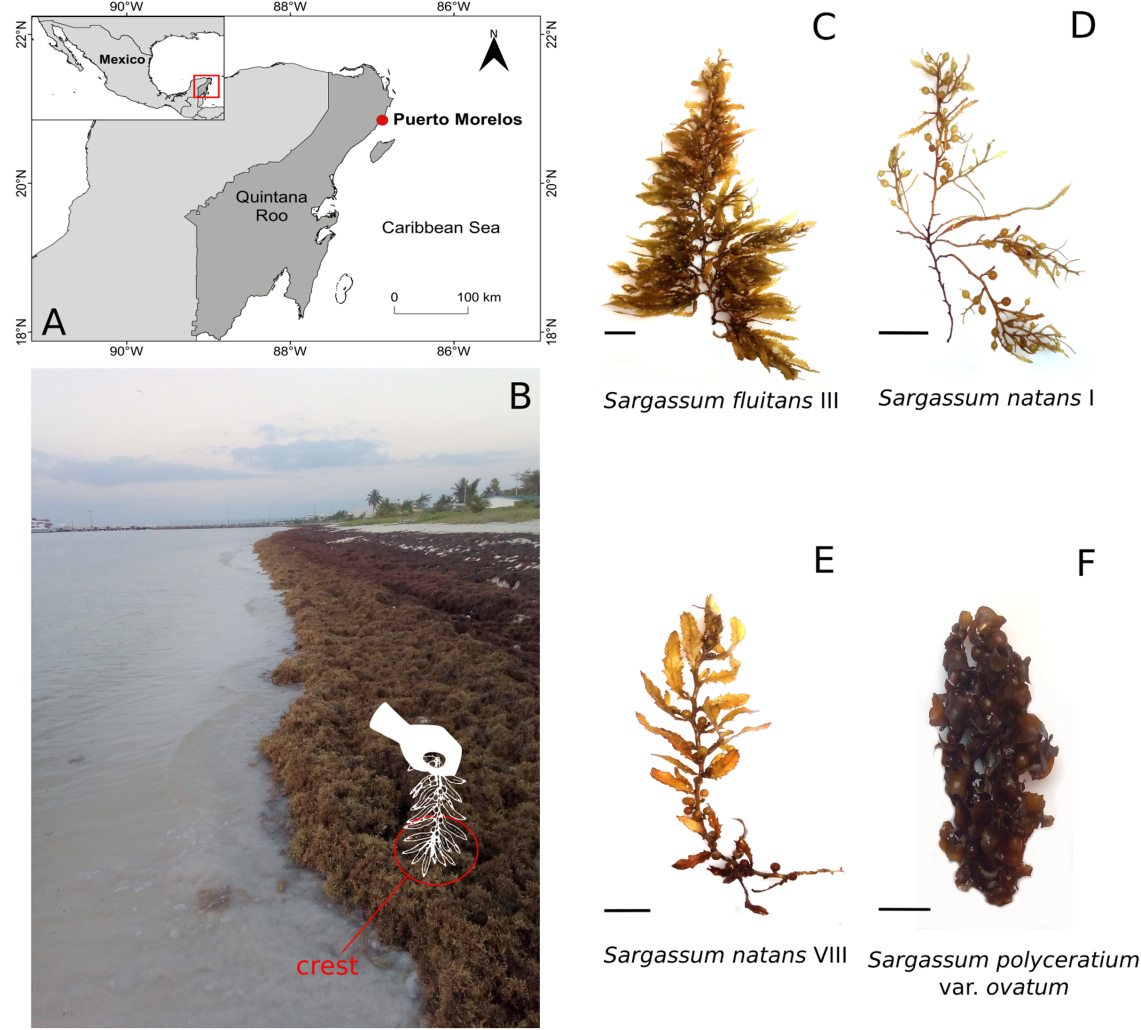
Study site. (A) Location of Puerto Morelos, Mexican Caribbean. (B) Specific location of sampling site. (C–F) *Sargassum* species collected. Scale bar = 2 cm.

Thalli of fresh pelagic *Sargassum* were collected on the beach at Puerto Morelos (Mexican Caribbean) (20.84 N–86.87 W) from April 2018 to March 2019 (Fig. 1A). Puerto Morelos is a small fishing and tourist village located on the northern part of an extensive barrier-fringing reef tract that extends from Belize to the Yucatan Strait. In the forereef zone, the most conspicuous components of the biota are gorgonians, small hemispherical coral heads and macroalgae (Ruíz-Rentería, Van Tussenbroek & Jordán-Dahlgren, 1998). The climate in the region has three characteristic seasons: warm and dry (March–May), winter storm with occasional short showers (November–February) and rainy (June–October) (Huntington, 1912; Schmitter-Soto et al., 2002). During the period of our study (2018–2019), temperature and rainfall data (values expressed as average) followed the same seasonal pattern: warm and dry (28.08 °C; 69.73 mm), winter storm (25.39 °C; 75.89 mm) and rainy (29.59 °C; 131.69 mm) (CONAGUA, 2018; 2019).

Each month, samples were taken from algal strandings on the beach, which remained wet by contact with waves. From each morphotype of *Sargassum*, a specimen with visible sessile epibionts was collected from the crest of the mounds of *Sargassum* (Fig. 1B). A total of 12 specimens were used to describe the occurrence and percent cover of hydroids on each *Sargassum* species and morphotypes. Thalli and epibionts were fixed in 96% ethanol. Algae in the samples were identified according to Parr (1939), Taylor (1960), Littler & Littler (2000) and Amaral-Zettler et al. (2017). Additionally, floating thalli of a local species of benthic *Sargassum*, incidentally detected in May and October 2018, were examined for epibiont hydroids.

**Figure 2:**
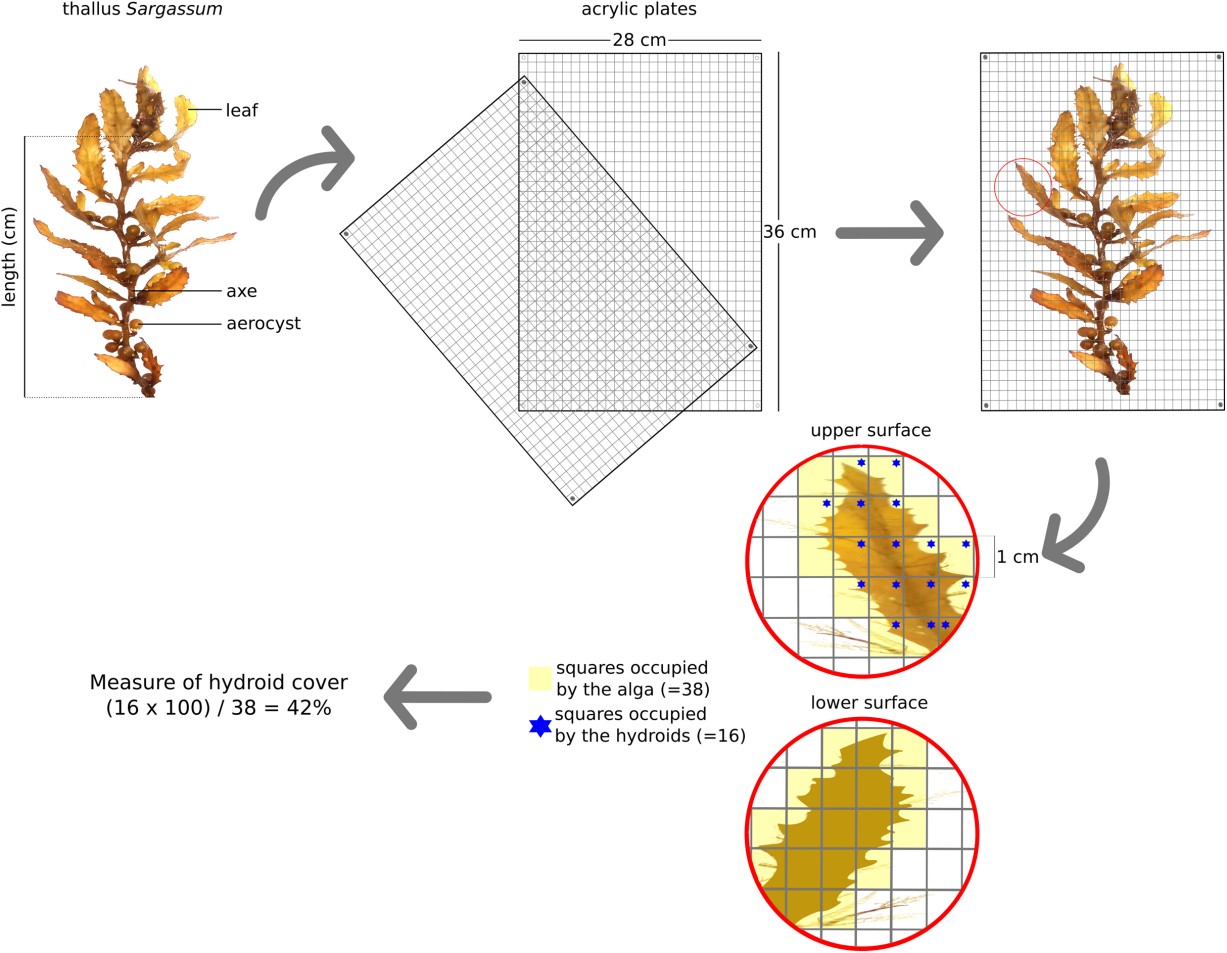
Scheme with details about methodology, with an example of the measure of hydroid cover found on the fronds of *Sargassum*. Adopted from the methodology described in Cunha & Jacobucci (2010).

In the laboratory, collected thalli were examined, and presence of hydrozoans in their axes, leaves and aerocysts was recorded. Hydroid coverage on thalli of both pelagic and benthic species of *Sargassum* was estimated following Cunha & Jacobucci (2010). The hydroid cover on each thallus (hydrorhiza of hydroids in contact with thallus) was measured by placing the alga between two clear rectangular acrylic plates, divided into 1 × 1 cm squares. When necessary, the thallus was cut into pieces to avoid the superposition of axes, leaves and aerocysts. Hydroid cover was estimated by counting the number of square divisions occupied by macroalgae with and without hydroids on both sides of the plates (Fig. 2).

Hydroids were identified to species level using morphological criteria in descriptions from the taxonomic literature (Calder, 1988, 1991b; Schuchert, 2012). Nomenclature used here generally follows that of the World Register of Marine Species (WoRMS, 2019) and Oliveira et al. (2016).

Non-parametric statistical analyses were performed to determine differences in species composition and percent cover of epibiont hydroids between pelagic *Sargassum* species and morphotypes using the Bray-Curtis similarity index. Species from the benthic alga were excluded from the statistical analyses because only two months of samples were available for study. Data matrices were standardized and transformed with square root; subsequently, a non-metric multidimensional scaling (nMDS) analysis was performed. To assess the graphical relationships plotted by nMDS analysis, we considered Clarke (1993), who provided an indicator of stress value: excellent graphical representation (<0.05), good ordination (<0.1), usable representation (<0.2), and a possible misinterpretation (>0.2). A similarity percentage (SIMPER) analysis was also performed to identify representative hydroid species of the different *Sargassum* species, and significant differences were evaluated using one-way analysis of similarity (ANOSIM) at a 0.1% significance level (Clarke & Warwick, 2001). All analyses were performed with the software PRIMER v6 (Clarke & Gorley, 2006).

Voucher specimens were deposited in the collection Cnidarians of the Gulf of Mexico and Mexican Caribbean “Lourdes Segura” (Faculty of Science, Multidisciplinary Teaching and Research Unit, Sisal, Yucatán, Mexico) under the code YUC–CC–254–11 from 001561 to 001569. The best preserved epibiont hydroids were selected for vouchering, with one of each species. Each specimen was deposited together with its fragment of *Sargassum*. These vouchers were indicated with a superindex in each of the taxa determined at the species level in Table 2 (indicated only by the six last numbers of the code).

## Results

Thalli of the specimens of *Sargassum* collected during the study corresponded to descriptions of the pelagic species and morphotypes of *Sargassum fluitans* III (*n* = 12; 19 ± 9 cm length; mean±SD), *S. natans* I (*n* = 12; 15 ± 4 cm length; mean ± SD) and *S. natans* VIII (*n* = 12; 23 ± 8 cm length; mean ± SD) and the benthic species *S. polyceratium* var. *ovatum* (Collins) W.R. Taylor (*n* = 2; 18 cm length; mean) (Figs. 1C–1F; Table 1).

**Table 1:** Data on pelagic and benthic *Sargassum* species from Puerto Morelos, Quintana Roo.

Year	Month	Size of thalli collected (cm)				Average of the percentage of hydroid cover				Number of epibiont hydroid taxa			
		Sf III	Sn I	Sn VIII	Spo	Sf III	Sn I	Sn VIII	Spo	Sf III	Sn I	Sn VIII	Spo
2018	April	16	15	22	nc	30.48	15.49	48.76	nc	2	3	1	nc
	May	29	10	28	18	47.89	15.38	55.63	49.71	1	1	1	2
	June	14	14	34	nc	47.62	7.08	59.75	nc	1	1	1	nc
	July	17	15	17	nc	30.38	0.53	79.70	nc	1	2	1	nc
	August	17	15	21	nc	9.26	0.00	50.70	nc	1	0	1	nc
	September	31	15	28	nc	29.34	24.16	38.16	nc	1	1	2	nc
	October	8	18	19	18	70.91	24.76	73.42	50.00	1	2	2	1
	November	19	10	16	nc	19.71	66.28	54.30	nc	2	1	1	nc
	December	10	13	17	nc	63.60	5.11	27.85	nc	1	2	3	nc
2019	January	11	13	17	nc	14.20	24.48	33.00	nc	2	1	2	nc
	February	21	13	13	nc	16.67	45.09	58.02	nc	3	2	1	nc
	March	38	24	38	nc	34.27	0.82	36.93	nc	1	4	1	nc
	Average	19	15	23	18	34.53	19.10	51.35	49.85	1.42	1.67	1.42	1.5

**Note:**

Sf III, *Sargassum fluitans* III; Sn I, *Sargassum natans* I; Sn VIII, *Sargassum natans* VIII; Spo, *Sargassum polyceratium* var. *ovatum*; nc, not collected.

**Figure 3:**
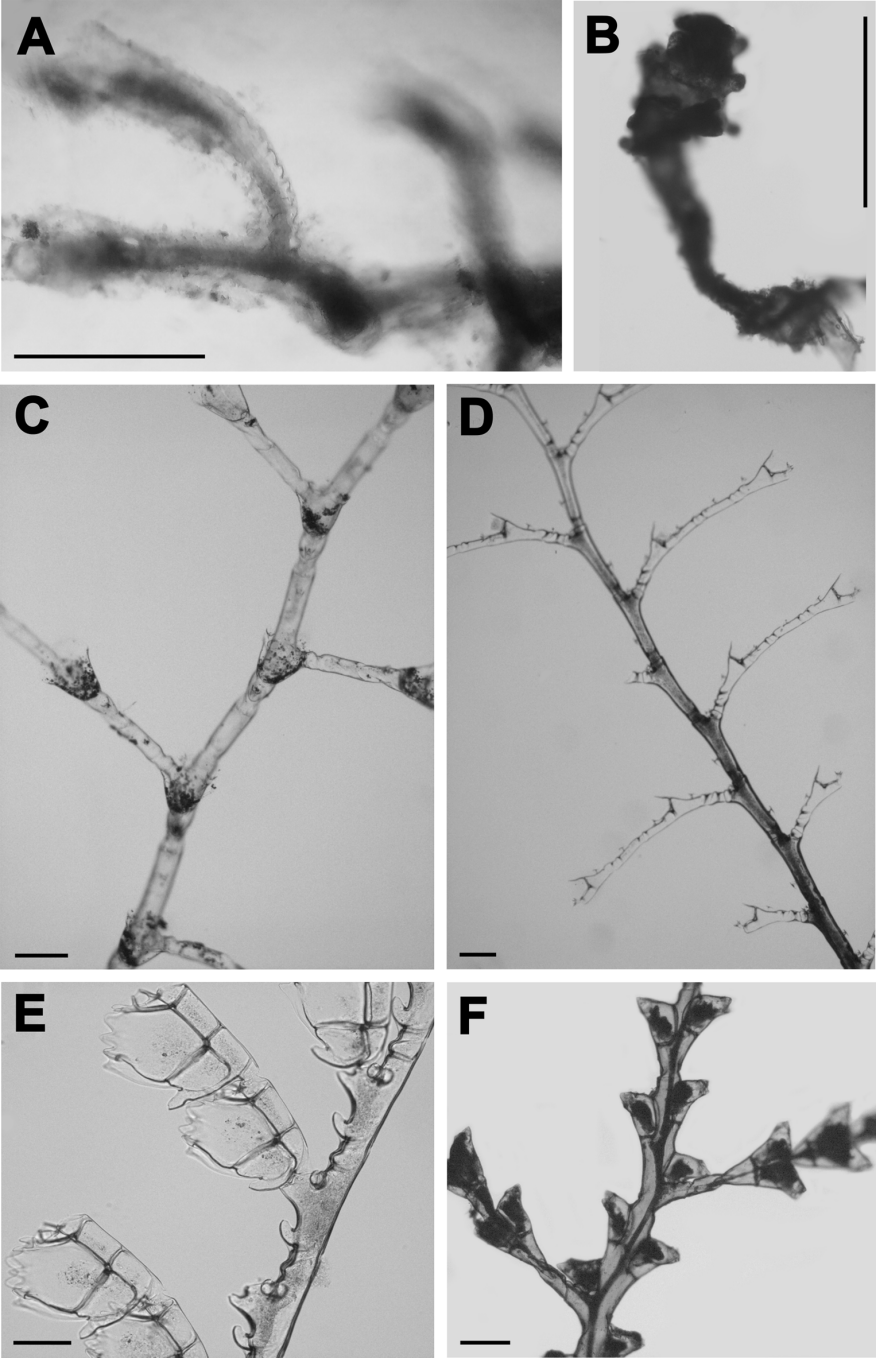
Epibiont hydroid species. (A and B) *Zanclea alba* (Meyen, 1834); (C) *Halopteris diaphana* (Heller, 1868); (D) *Plumularia strictocarpa* Pictet, 1893; (E) *Aglaophenia latecarinata* Allman, 1877; (F) *Tridentata marginata* (Kirchenpauer, 1864). Scales for (A), (B), (D) and (E) equal 0.15 mm, for (C) and (F) equal 0.3 mm.

**Figure 4:**
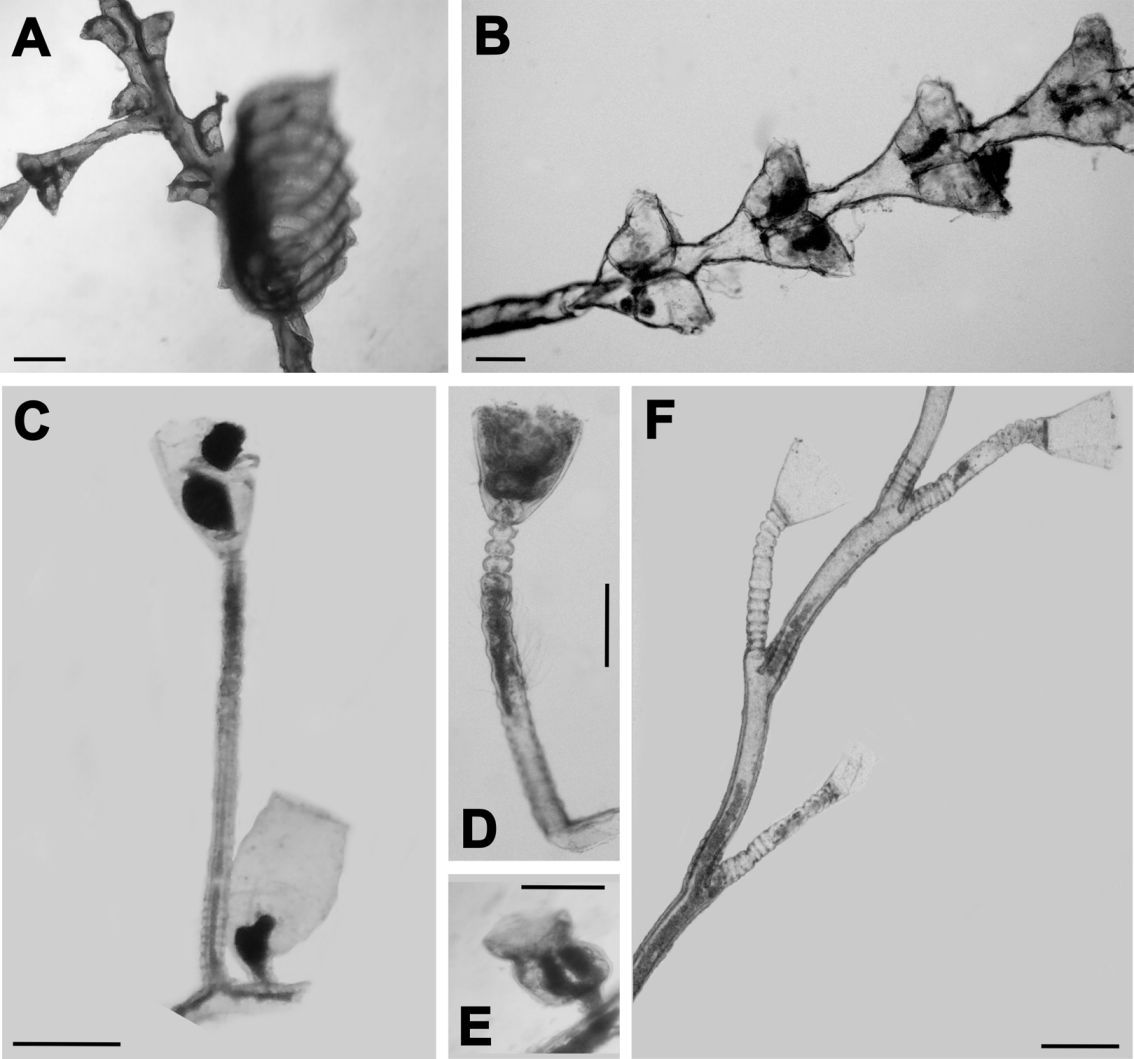
Epibiont hydroid species. (A) *Tridentata marginata* (Kirchenpauer, 1864); (B) *Tridentata turbinata* (Lamouroux, 1816); (C) *Clytia hemisphaerica* (Linnaeus, 1767); (D and E) *Clytia noliformis* (McCrady, 1859); (F) *Obelia dichotoma* (Linnaeus, 1758). Scales from (A) to (F) equal 0.3 mm.

Throughout the entire annual cycle, 14 epibiont taxa of hydroids were recorded. Nine of these were identified to species (Figs. 3 and 4), comprising seven genera, seven families, three orders and two superorders (“Anthoathecata”, which is non-monophyletic and Leptothecata) (Table 2). Due to damaged and insufficient morphological diagnostic characteristics in some specimens, three taxa were identified to genus (*Halopteris*, *Clytia*, *Obelia*), one to family (Plumulariidae) and one to suborder (Proboscoida). In addition to hydroids, bryozoans, mainly *Jellyella tuberculata*, and Polychaeta (serpulid worms) were observed on *Sargassum* thalli. Of the epibiont hydroid taxa associated with *Sargassum* thalli, 13 were observed on the axes, 12 on leaves and eight on aerocysts. *Obelia dichotoma* was found only on thalli axes, while *Halopteris diaphana* and *Tridentata marginata* were not found on thalli aerocysts (Table 2).

**Table 2:** Occurrence (% cover) of hydroid species on pelagic *Sargassum* species and morphotypes, and on benthic *S. polyceratium *var. *ovatum* from Puerto Morelos, Quintana Roo.

Taxon	Annual average of the percentage of cover	*Sargassum* species	*Sargassum* regions with hydroids			Previous records in pelagic and benthic *Sargassum* species
			Axes	Leaves	Aerocysts	
Superorder “Anthoathecata” Cornelius, 1992						
Order Capitata Kühn, 1913						
Zancleidae Russell, 1953						
*Zanclea alba* (Meyen, 1834)^001569^	1.33	Sn I	*x*	*x*	*x*	Sf, Sn (4)
	0.36	Sn VIII	*x*	*x*		
Superorder Leptothecata Cornelius, 1992						
Order Macrocolonia Leclère, Schuchert, Cruaud, Couloux & Manuel, 2009						
Suborder Plumupheniida Maronna, Miranda, Peña Cantero, Barbeitos & Marques, 2016						
Halopterididae Millard, 1962						
*Halopteris diaphana* (Heller, 1868)^001564^	0.32	Sf III	*x*	*x*		Sc (9); Sfu (10); Sf, Sn (4)
	2.32	Sn I	*x*	*x*		
	0.17	Sn VIII	*x*			
*Halopteris* spp.	1.28	Sn I	*x*			
Plumulariidae McCrady, 1859	0.03	Sf III	*x*			
*Plumularia strictocarpa* Pictet, 1893^001566^	1.64	Sf III	*x*	*x*	*x*	Sc (9); Sf, Sn (4); Sh, St, Sto (2); Sfu (8)
	0.92	Sn I	*x*	*x*	*x*	
Aglaopheniidae Marktanner-Turneretscher, 1890						
*Aglaophenia latecarinata* Allman, 1877^001561^	62.24	Sn VIII	*x*	*x*	*x*	Sc (9, 10); Sf (4); Sf III (11); Sfu (10), Sn I (11); Sn VIII (11)
Suborder Sertulariida Maronna, Miranda, Peña Cantero, Barbeitos & Marques, 2016						
Sertulariidae Lamouroux, 1812.						
*Tridentata marginata* (Kirchenpauer, 1864)^001567^	46.00*	Spo	*x*	*x*		Ss (6); Sc (8, 9, 10)
*Tridentata turbinata* (Lamouroux, 1816)^001568^	28.70*	Spo	*x*	*x*	*x*	Sc (8, 9, 10); Sfu (8, 10)
Order Statocysta Leclère, Schuchert, Cruaud, Couloux & Manuel, 2009						
Suborder Proboscoida Broch, 1910	3.99	Sf III	*x*	*x*		
	0.06	Sn I	*x*			
Clytiidae Cockerell, 1911						
*Clytia hemisphaerica* (Linnaeus, 1767)^001562^	5.0	Sn VIII	*x*	*x*	*x*	Sa (5); Sf, Sn (4); Ss (6); Sc, Sfu (8)
*Clytia noliformis* (McCrady, 1859)^001563^	36.63	Sf III	*x*	*x*	*x*	Sf X (1); Sf (4); Sfu (8, 10); Si (7); Sn I, Sn II, Sn VIII, Sn IX (1); Sn (3, 4); Ssw (7)
	15.08	Sn I	*x*	*x*	*x*	
	0.22	Sn VIII		*x*		
*Clytia* spp.	5.52	Sn I	*x*	*x*	*x*	
Obeliidae Haeckel, 1879						
*Obelia dichotoma* (Linnaeus, 1758)^001565^	0.06	Sf III	*x*			Sh (2); Sa (5); Sc (8, 9, 10); Sfu (8, 10)
	1.07	Sn I	*x*	*x*	*x*	
*Obelia* spp.	0.59	Sn I		*x*		

**Notes:**

* Average of the percentage of cover, only considering the two months in which *S. polyceratium* var. *ovatum* was collected. Superindex indicate the catalog number of epibiont hydroids deposited in the collection.

Sa, *Sargassum acinarium* (Linnaeus) Setchell; Sc, *Sargassum cymosum* C. Agardh; Sf, *Sargassum fluitans* (Bøergesen) Bøergesen; Sf III, *Sargassum fluitans* III; Sfu, *Sargassum* cf. *furcatum* Kutzing; Sh, *Sargassum hemiphyllum* (Turner) C. Agardh; Si, *Sargassum ilicifolium* (Turner) C. Agardh; Sn, *Sargassum natans* (Linnaeus) Gaillon; Sn I, *Sargassum natans* I; Sn II, *Sargassum natans* II; Sn VIII, *Sargassum natans* VIII; Sn IX, *Sargassum natans* IX; Spo, *Sargassum polyceratium* var. *ovatum* (Collins) W.R. Taylor; Ss, *Sargassum stenophyllum* (undefined authority); Ssw, *Sargassum swartzii* C.Agardh; St, *Sargassum thunbergii* (Merten ex Roth) Kuntze; Sto, *Sargassum tortile* (C. Agardh) C. Agardh. (1) Parr (1939), (2) Nishihira (1965), (3) Ryland (1974), (4) Calder (1995), (5) Morri & Bianchi (1999), (6) Haddad & Chiaverini (2000), (7) Moazzam & Moazzam (2006), (8) Oliveira, Marques & Migotto (2006), (9) Cunha & Jacobucci (2010), (10) Oliveira & Marques (2011) and (11) Govindarajan et al. (2019). *x*, presence.

Two epibiont hydroid species were recorded on benthic *S. polyceratium* var. *ovatum*, which are new records for this algal species (Table 2). *Tridentata marginata* was present in May and October 2018, while *T. turbinata* was observed only in May 2018. The average cover of these two hydrozoans, only considering the two months in which *S. polyceratium* var. *ovatum* was collected, was 37.35%.

**Figure 5:**
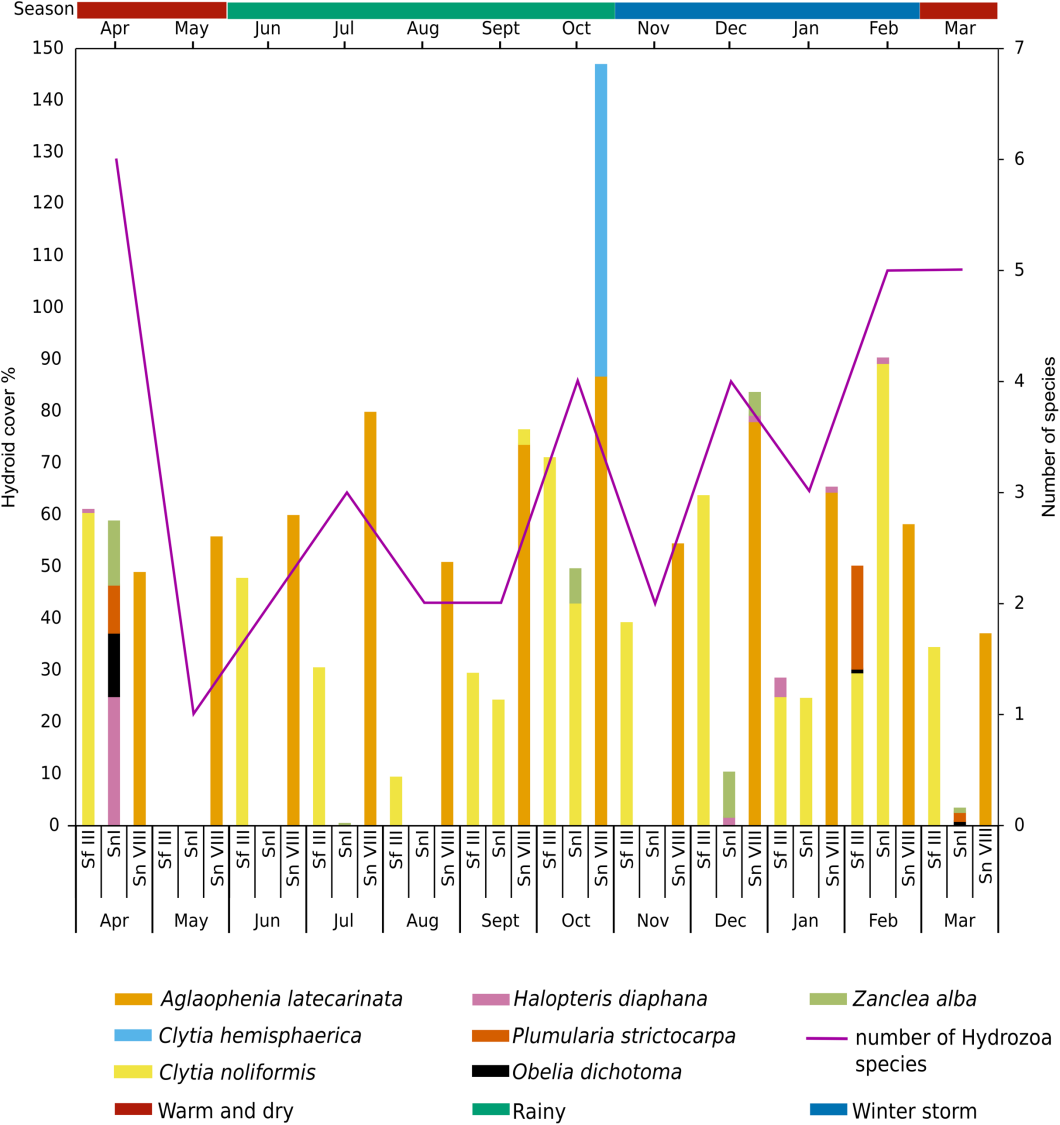
Seasonal and monthly hydroid cover (%) on pelagic *Sargassum* species and morphotypes collected from Puerto Morelos, Quintana Roo, Mexico, from April 2018 to March 2019. Sf III, *Sargassum fluitans* III; Sn I, *Sargassum natans* I; Sn VIII, *Sargassum natans* VIII. Season; hydroid cover (%). Each bar represents the sum of each hydroid species cover (%). The purple line represents the monthly number of hydrozoan species on all pelagic species of *Sargassum*. Colors optimized for color-blind individuals according to Wong (2011).

Over the year of study, four epibiont hydroid species were recorded on *S. fluitans* III, five on *S. natans* I and five on *S. natans* VIII (Table 2). We found that *Aglaophenia latecarinata* thrives year-round, followed by *C. noliformis*, which was recorded 11 of 12 months (in all seasons). *Clytia hemisphaerica* was recorded only in the month of October (rainy season) (Fig. 5). Reproductive structures were noted in *C. hemisphaerica* and *C. noliformis* (September, October and December), during the transition from the rainy season to winter storm season. Combining monthly data on hydroids from pelagic morphotypes of *Sargassum* during the study, highest overall species richness of hydroids was recorded in April (six species), followed by February and March (five species), followed by a marked decrease in May (one species), the end of the warm and dry season (Fig. 5).

**Figure 6:**
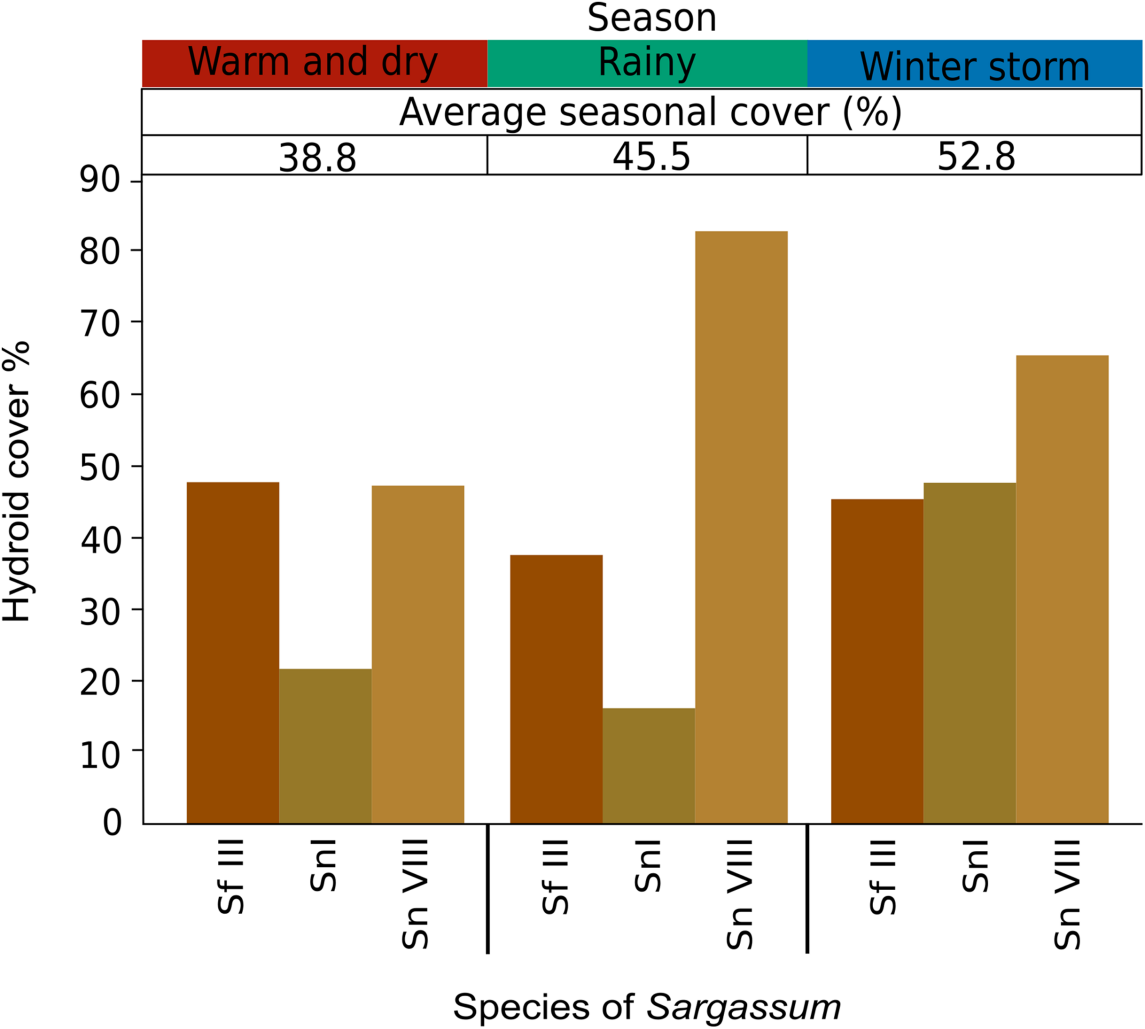
Average seasonal and monthly cover (%) of epibiont hydroid species on pelagic *Sargassum* species and morphotypes collected from Puerto Morelos, Quintana Roo, Mexico, from April 2018 to March 2019. Sf III, *Sargassum fluitans* III; Sn I, *Sargassum natans* I; Sn VIII, *Sargassum natans* VIII. Colors optimized for color-blind individuals according to Wong (2011).

The annual average cover of hydrozoans on *S. fluitans* III, *S. natans* I and *S. natans* VIII were 42.67%, 28.17% and 67.97%, respectively. *Clytia noliformis* and *A. latecarinata* were the predominant species, accounting for 36.63% on *S. fluitans* III and 62.24% on *S. natans* VIII. Notably, *A. latecarinata* and *C. hemisphaerica* were recorded exclusively on *S. natans* VIII (Table 2). Throughout the year, the percentage cover of hydrozoan epibionts on species of pelagic *Sargassum* was higher in the winter storm season (52.80%) (Fig. 6). Further seasonal breakdown shows that the percentage occupation of epibiont hydroids on structures of pelagic *Sargassum* species was highest (95.24%) on the axes during the winter storm season and lowest (47.06%) on the aerocysts during the warm and dry season.

**Figure 7:**
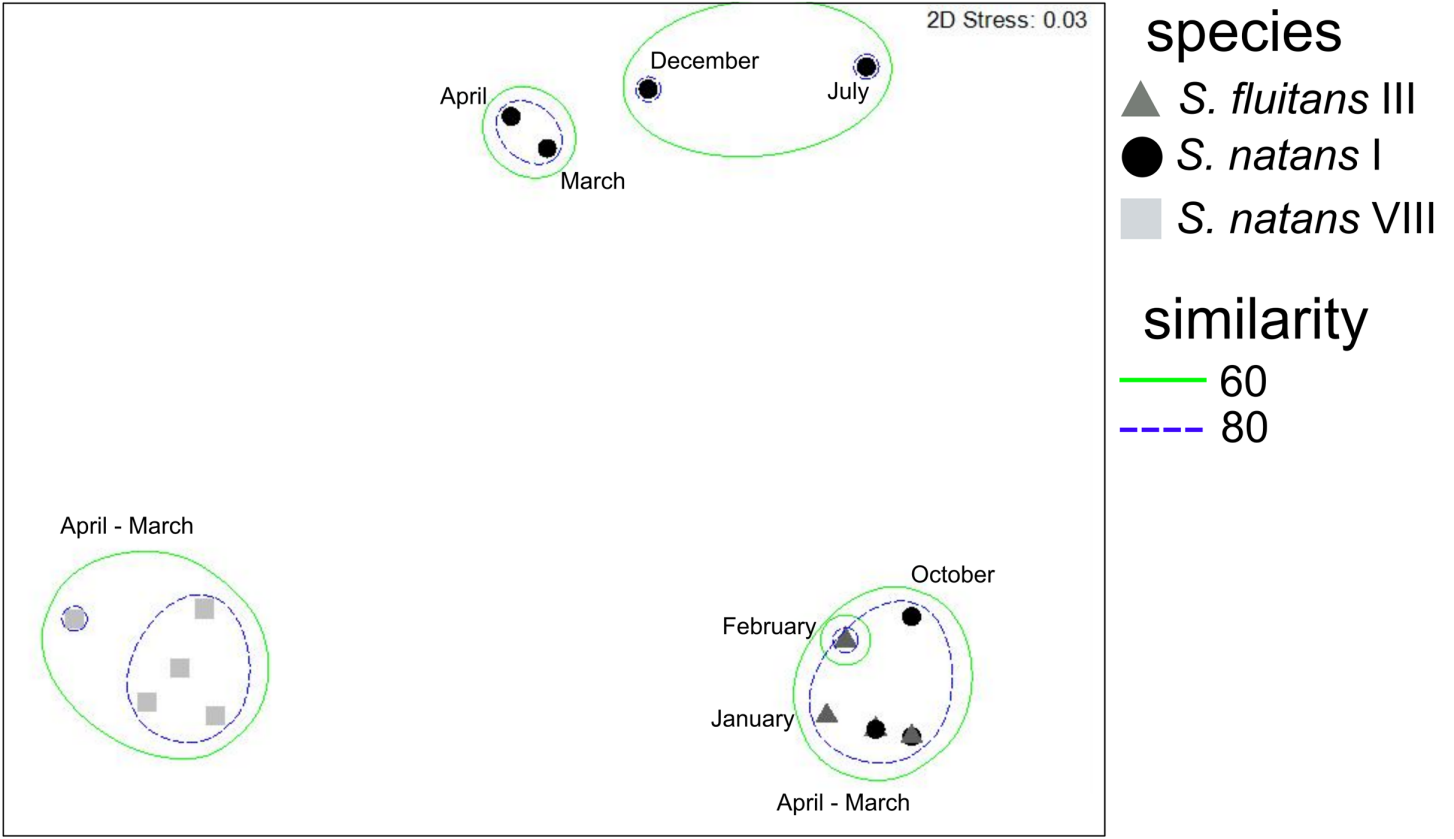
Non-metric multidimensional scaling (nMDS) plot based on Bray-Curtis similarity of hydroid cover (%) on pelagic *Sargassum* species and morphotypes (April 2018–March 2019).

The nMDS ordination displays the spatial distribution of the monthly pelagic *Sargassum* species and morphotypes samples and a clear separation into three groups of epibiont hydroids. The stress value obtained with the ordination was 0.03 (Fig. 7). These groupings were significantly different based on the ANOSIM (global *R* = 0.78; *P* < 0.001) analysis. When compared by pairs, those with significant differences between them were *S*. *natans* VIII and *S. fluitans* III (*R* = 1; *P* < 0.001) and *S*. *natans* VIII and *S. natans* I (*R* = 0.88; *P* < 0.001), while no significant differences were found between *S. natans* I and *S. fluitans* III (*R* = 0.33; *P* > 0.001).

The SIMPER analysis showed that hydroids present on *S. fluitans* III had 89.32% similarity, while those on *S. natans* I showed a similarity of 34.79%, and those on *S. natans* VIII 89.26%. The hydroid *A. latecarinata* makes the most important contribution to the similarity of the hydroid assemblage (99.85%) on *S. natans* VIII, while variations in the percentage cover of hydroids of *C. noliformis* characterized assemblages on *S. fluitans* III (99.85%) and *S. natans* I (54.49%). *Zanclea alba* also makes an important contribution (33.21%) to the similarity in *S. natans* I assemblages (Table 3).

**Table 3:** Contribution of hydroid species to the observed similarity between pelagic *Sargassum* species and morphotypes calculated by similarity percentages (SIMPER).

Groups	Av. cov.	Av. sim.	Sim/SD	Contrib. %	Cum. %
*Sargassum fluitans* III					
Average similarity: 89.32					
*Clytia noliformis*	9.73	89.19	5.87	99.85	99.85
*Sargassum natans* I					
Average similarity: 34.79					
*Clytia noliformis*	4.91	18.96	0.51	54.49	54.49
*Zanclea alba*	3.97	11.55	0.63	33.21	87.70
*Halopteris diaphana*	1.77	2.71	0.44	7.79	95.49
*Sargassum natans* VIII					
Average similarity: 89.26					
*Aglaophenia latecarinata*	9.76	89.13	6.60	99.85	99.85

**Note:**

Av. cov., average hydroid cover; Av. sim., average similarity; Sim/SD, similarity to standard deviation ratio; Contrib %, percentage contribution; Cum. %, cumulative percentage contribution.

## Discussion

This study examined the species richness, occurrence, percentage cover and variations in composition of epibiont hydroids occurring on species of *Sargassum*. Of those studied, 92.86% were thecate hydroid taxa (Table 2). All taxa of hydroids identified during this study have been recorded earlier in the Gulf of Mexico (Calder & Cairns, 2009; Mendoza-Becerril, Simões & Genzano, 2018). However, these taxa cannot be considered as new records for the study area, because the local benthic hydroid species are unknown, and records here are of hydroids on drifting substrates. As suggested by Putman et al. (2018), pelagic *Sargassum* is transported through the open ocean and Caribbean Sea following several pathways until it reaches the Caribbean coast of Mexico.

The two hydroids occurring on benthic *S. polyceratium* var. *ovatum*, a species forming part of the local flora of Puerto Morelos (Díaz-Martín & Espinoza-Avalos, 2000), were not observed on the pelagic species and morphotypes of *Sargassum* species; this suggests that they may also be part of the local hydroid fauna. However, these hydroids have been recorded from pelagic (Stachowicz & Lindquist, 1997) and benthic species (Table 2). These hydroid species are new records from *S. polyceratium* var. *ovatum*. Nevertheless, it will be necessary to perform systematic surveys of both pelagic and benthic species of *Sargassum* to confirm if these hydroid species are exclusively found on *S. polyceratium* var. *ovatum* in the region.

The number of epibiont species found on beachcast *Sargassum* in Puerto Morelos (from two to five hydrozoan species per macroalgal species and morphotypes) is relatively low compared with other studies of the same algal genus. Nishihira (1965) recorded eight hydrozoan species on *Sargassum hemiphyllum* (Turner) C. Agardh from Japan, while Calder (1995) reported 10 species for *S. fluitans* III and eight species for *S. natans* in Bermuda. Moreover, Cunha & Jacobucci (2010) found 14 hydroid species on *S. cymosum* from Brazil. From all species of hydroids recorded on *Sargassum* here and in other cited studies, three are most frequent: *A. latecarinata*, *C. noliformis* and *O. dichotoma* (Table 2). Parr (1939) and Govindarajan et al. (2019) mentioned that *A. latecarinata* is often found on *S. fluitans* III and *S. natans* VIII.

We observed more hydroid taxa growth on axes compared with leaves and aerocysts. This comparison is the first at structural level of thallus, since other studies describe the variation in hydroid colonization along the thallus. For example, Fletcher & Day (1983) mentioned that the abundance of hydroids decreases distally in brown macroalgae *Ecklonia radiata* (C. Agardh) J. Agardh. Faucci & Boero (2000) reported 86% abundance of hydroids on the basal part of brown macroalgae *Cystoseira* spp., and Fraschetti et al. (2006) found significant differences in growth among thallus regions (basal, medium or distal parts), with the highest number of taxa often recorded on the basal portion of *Cystoseira*. This variation has been attributed to competitive interactions between the component species within the community or interactions between the epifauna and the host algae (Seed, 1986), growth rate of the epibiont and host (Rackley, 1974) and exposure to mechanical stress (e.g., abrasion, friction, water movement) (Faucci & Boero, 2000).

Our data no suggest seasonal variations in epibiont hydroid species richness, but were noted a variation in terms of percentage cover. Cunha & Jacobucci (2010) recorded seasonal variations, which tend to be higher during the Brazilian summer. Such seasonal variations have been attributed to fluctuations of biotic and abiotic factors (c.f. Cunha & Jacobucci, 2010; Cunha, Maruyama & Jacobucci, 2018), including macroalgal growth periods and/or hydroid reproductive strategies. Nonetheless, to clarify and contribute to a better understanding of any seasonal variation, supplementary studies are essential, both of the epibionts and their living substrates, and the types of specific interactions between them.

The three groups of epibiont hydroids characteristic of the pelagic *Sargassum* species and morphotypes had an excellent graphical representation and a strong relationship between epibiont-substrate (stress value = 0.03; global *R* = 0.78; *P* < 0.001). Differences in hydroid species composition on species and morphotypes of *Sargassum* may be associated with algal morphology and/or the substances they produce (Nishihira, 1967, 1968), as well as the growth patterns of *Sargassum* and its epibionts (Burkenroad in Parr (1939)). Similar differences have been found in previous studies of floating *Sargassum* masses in the Sargasso Sea (Parr, 1939; Niermann, 1986; Calder, 1995). However, the hydroid taxa reported here are not exclusively found in *Sargassum*, as these hydroids have also been recorded on floating objects, rocks and other benthic macroalgae (Calder, 1995; Oliveira & Marques, 2007; Oliveira & Marques, 2011).

Finally, it should be noted that while hydroids on beachcast *Sargassum* were often deteriorated, it was possible to find colony parts with intact coenosarc in thecate species. Deterioration may be due to being fixed to a mobile and flexible substrate, to the mechanical stresses related to the action of currents before reaching land, to abrasion by surf and sand particles as they strand on beaches, and to exposure to air and terrestrial weather once ashore. Whatever the reason for the deterioration, and as suggested by Riedl (1966) and Gili et al. (1998), only hydroids with chitinous exoskeletons, including hydrothecae (superorder Leptothecata) usually survive such adverse environmental conditions. In other studies on hydroids of pelagic *Sargassum*, leptothecate taxa dominate, with percentages above 80% (Calder, 1995; Martinelli-Filho, Morais & Aviz, 2016; Mendoza-Becerril, Simões & Genzano, 2018). Given the regenerative potential of hydroids, attachment of colonies on autochthonous substrates can occur. Moreover, nematocysts were observed in collected material, indicating that hydroids maintain their stinging capacity and the ability to cause painful rashes and erythematous papules, as has observed in other species of Hydrozoa (Rifkin, Fenner & Williamson, 1993; Marques, Haddad & Migotto, 2002).

In addition, studies on the hydroids of *Sargassum* are warranted because these invertebrates are effective invaders (Ma & Purcell, 2005) and may be co-introduced on substrates such as seaweeds (Cornelius, 1981; Martinelli-Filho, Morais & Aviz, 2016), which can have different origins, as mass strandings of *Sargassum* (Sissini et al., 2017). This study recorded the presence of *O. dichotoma*, which has been considered an invasive species in Mexico (González et al., 2014; Global Biodiversity Information Facility (GBIF), 2019) and a common epibiont hydroid of pelagic and benthic *Sargassum* (six species) (Nishihira, 1965; Calder, 1995; Morri & Bianchi, 1999; Oliveira, Marques & Migotto, 2006; Cunha & Jacobucci, 2010). We also recorded thriving colonies of *A. latecarinata*, which it is an aglaophenid no venomous to humans, contrary to other members of the family Aglaopheniidae (e.g., *A. cupressina, A. pluma, M. philippina*) (Svoboda & Cornelius, 1991; Tang, 1991, as cited in Tseng et al., 2014; Rifkin, Williamson & Fenner, 1996; Santhanam, 2020).

Inasmuch as reports on major strandings of *Sargassum* in the Caribbean region are relatively recent, advance knowledge of these events beyond the adverse effects is needed (c.f. Hinds et al., 2016; Dutch Carribean Nature Alliance, 2019). The impacts of epibionts (e.g., hydroids) on seaweeds need to be assessed along with the phenomenon of golden tides. Awareness of the impact of toxic sessile and motile biota on humans will be needed as recommendations for the use and management of beachcast *Sargassum* are promulgated.

## Conclusions

The coastal area of the Mexican Caribbean has recently experienced massive incursions of brown seaweeds. The epibiont fauna of these phyophyceans is entirely unknown here, and its origin has not been determined, whether local or transported from other regions along with their substrates. This study provides the first report of epibiont hydroid taxa on certain species and morphotypes of beachcast pelagic *Sargassum*. Moreover, data are provided on their occurrence and percent coverage, and distribution among axes, leaves and aerocysts of the algal thalli. The composition and occurrence of hydroids changes monthly and varies between *Sargassum* species and their morphotypes.

## Supplemental Information

10.7717/peerj.9795/supp-1 Supplemental Information 1 Raw data employed to calculate the percentage cover of hydroid species in pelagic Sargassum species and morphotypes and in benthic *S. polyceratium* var. ovatum.

10.7717/peerj.9795/supp-2 Supplemental Information 2 Results of the similarity percentages analysis between pelagic Sargassum species and morphotypes.

10.7717/peerj.9795/supp-3 Supplemental Information 3 Raw data employed and results to obtain the ANOSIM between pelagic Sargassum species and morphotypes.

## Additional Information and Declarations

### Data Availability

The following information was supplied regarding data availability: Raw data is available in the Supplemental Files.
