# Supplementary material for: Epibiont hydroids on beachcast Sargassum in the Mexican Caribbean
Source: PeerJ. 2020 Aug 24;8:e9795. doi: 10.7717/peerj.9795 (PMC7450996; doi:10.7717/peerj.9795)
Supplement: Supplemental Information 2 [file peerj-08-9795-s002.rtf]

SIMPER
Similarity Percentages - species contributions

One-Way Analysis

Data worksheet
Name: Data2
Data type: Other
Sample selection: All
Variable selection: All

Parameters
Resemblance: S17 Bray Curtis similarity
Cut off for low contributions: 90.00%

Factor Groups
Sample	Especie
april SF	SF
june SF	SF
july SF	SF
august SF	SF
september SF	SF
october SF	SF
november SF	SF
december SF	SF
january SF	SF
february SF	SF
march SF	SF
april SN	SN
july SN	SN
september SN	SN
october SN	SN
december SN	SN
january SN	SN
february SN	SN
march SN	SN
april SN(VIII)	SN(VIII)
may SN(VIII)	SN(VIII)
june SN(VIII)	SN(VIII)
july SN(VIII)	SN(VIII)
august SN(VIII)	SN(VIII)
september SN(VIII)	SN(VIII)
october SN(VIII)	SN(VIII)
november SN(VIII)	SN(VIII)
december SN(VIII)	SN(VIII)
january SN(VIII)	SN(VIII)
february SN(VIII)	SN(VIII)
march SN(VIII)	SN(VIII)

Group SF
Average similarity: 89.32

Species	Av.Abund	Av.Sim	Sim/SD	Contrib%	Cum.%
Clytia noliformis 	    9.73	 89.19	  5.87	   99.85	99.85

Group SN
Average similarity: 34.79

Species	Av.Abund	Av.Sim	Sim/SD	Contrib%	Cum.%
Clytia noliformis 	    4.91	 18.96	  0.51	   54.49	54.49
Zanclea alba	    3.97	 11.55	  0.63	   33.21	87.70
Halopteris diaphana	    1.77	  2.71	  0.44	    7.79	95.49

Group SN(VIII)
Average similarity: 89.26

Species	Av.Abund	Av.Sim	Sim/SD	Contrib%	Cum.%
Aglaophenia latecarinata	    9.76	 89.13	  6.60	   99.85	99.85

Groups SF  &  SN
Average dissimilarity = 53.46

	Group SF	Group SN	       	       	        	     
Species	Av.Abund	Av.Abund	Av.Diss	Diss/SD	Contrib%	Cum.%
Clytia noliformis 	    9.73	    4.91	  19.95	   1.03	   37.31	37.31
Zanclea alba	    0.00	    3.97	  16.49	   0.96	   30.85	68.17
Halopteris diaphana	    0.39	    1.77	   6.93	   0.89	   12.96	81.12
Plumularia strictocarpa	    0.57	    1.41	   6.09	   0.63	   11.39	92.52

Groups SF  &  SN(VIII)
Average dissimilarity = 98.36

	Group SF	Group SN(VIII)	       	       	        	     
Species	Av.Abund	      Av.Abund	Av.Diss	Diss/SD	Contrib%	Cum.%
Aglaophenia latecarinata	    0.00	          9.76	  45.65	   7.77	   46.41	46.41
Clytia noliformis 	    9.73	          0.15	  44.88	   6.60	   45.62	92.03

Groups SN  &  SN(VIII)
Average dissimilarity = 97.78

	Group SN	Group SN(VIII)	       	       	        	     
Species	Av.Abund	      Av.Abund	Av.Diss	Diss/SD	Contrib%	Cum.%
Aglaophenia latecarinata	    0.00	          9.76	  41.76	   5.65	   42.70	42.70
Clytia noliformis 	    4.91	          0.15	  22.65	   1.01	   23.17	65.87
Zanclea alba	    3.97	          0.19	  16.33	   0.98	   16.70	82.57
Halopteris diaphana	    1.77	          0.19	   6.57	   0.86	    6.72	89.29
Plumularia strictocarpa	    1.41	          0.00	   4.72	   0.54	    4.82	94.12
