# Supplementary material for: Epibiont hydroids on beachcast Sargassum in the Mexican Caribbean
Source: PeerJ. 2020 Aug 24;8:e9795. doi: 10.7717/peerj.9795 (PMC7450996; doi:10.7717/peerj.9795)
Supplement: Supplemental Information 3 [file peerj-08-9795-s003.rtf]

ANOSIM
Analysis of Similarities

One-Way Analysis

Resemblance worksheet
Name: Resem1
Data type: Similarity
Selection: All

Factor Values
Factor: Especie
SF
SN
SN(VIII)

Factor Groups
Sample	Especie
april SF	SF
june SF	SF
july SF	SF
august SF	SF
september SF	SF
october SF	SF
november SF	SF
december SF	SF
january SF	SF
february SF	SF
march SF	SF
april SN	SN
july SN	SN
september SN	SN
october SN	SN
december SN	SN
january SN	SN
february SN	SN
march SN	SN
april SN(VIII)	SN(VIII)
may SN(VIII)	SN(VIII)
june SN(VIII)	SN(VIII)
july SN(VIII)	SN(VIII)
august SN(VIII)	SN(VIII)
september SN(VIII)	SN(VIII)
october SN(VIII)	SN(VIII)
november SN(VIII)	SN(VIII)
december SN(VIII)	SN(VIII)
january SN(VIII)	SN(VIII)
february SN(VIII)	SN(VIII)
march SN(VIII)	SN(VIII)

Global Test
Sample statistic (Global R): 0.784
Significance level of sample statistic: 0.1%
Number of permutations: 999 (Random sample from a large number)
Number of permuted statistics greater than or equal to Global R: 0

Pairwise Tests
	        R	Significance	    Possible	      Actual	Number >=
Groups	Statistic	     Level %	Permutations	Permutations	 Observed
SF, SN	    0.337	         0.6	       75582	         999	        5
SF, SN(VIII)	        1	         0.1	     1352078	         999	        0
SN, SN(VIII)	    0.881	         0.1	      125970	         999	        0

Outputs
Plot: Graph9
